# Supplementary material for: White-nose syndrome restructures bat skin microbiomes
Source: Microbiol Spectr. 2023 Oct 27;11(6):e02715-23. doi: 10.1128/spectrum.02715-23 (PMC10714735; doi:10.1128/spectrum.02715-23)
Supplement: Figure S1 — 16S and ITS alpha rarefaction plots. [file spectrum.02715-23-s0001.pdf]

(a)

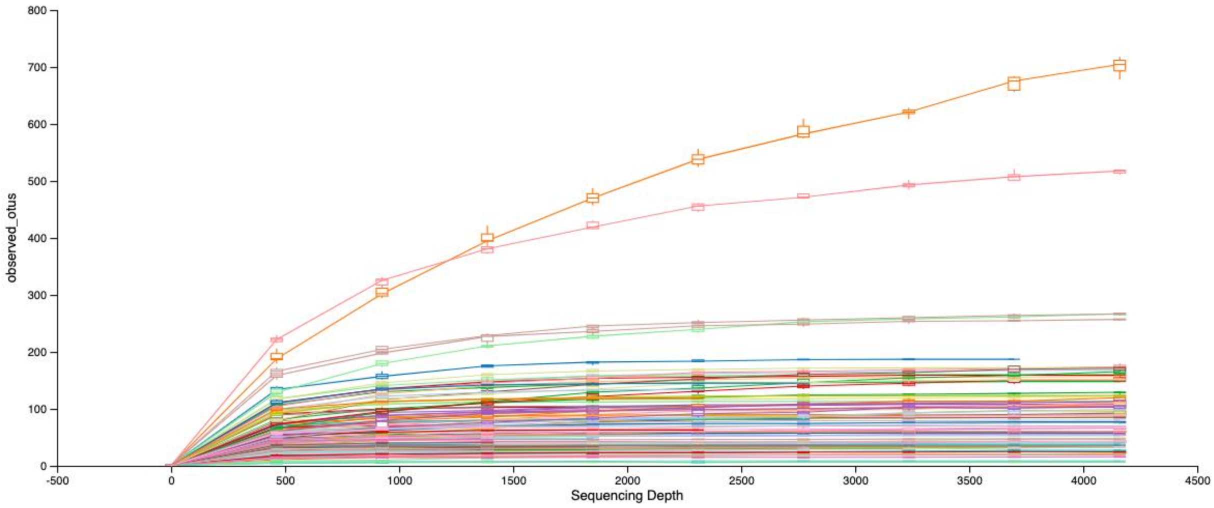

(b)

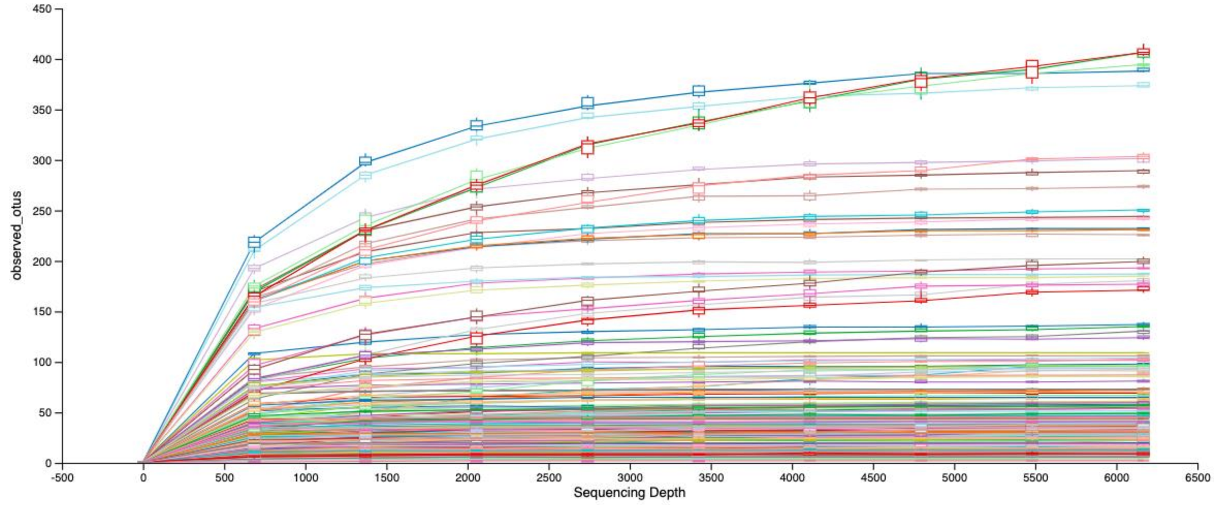

Figure S1. (a) 16S rRNA alpha rarefaction plot. An alpha rarefaction plot indicated no increase in the number of observed bacterial OTUs with increased sequencing depth for all but two samples when rarefied to 4,160 reads per sample. (b) ITS alpha rarefaction plot. An alpha rarefaction plot indicated no increase in the number of observed fungal OTUs with increased sequencing depth for all samples when rarefied to 6,167 reads per sample.
