# Supplementary material for: White-nose syndrome restructures bat skin microbiomes
Source: Microbiol Spectr. 2023 Oct 27;11(6):e02715-23. doi: 10.1128/spectrum.02715-23 (PMC10714735; doi:10.1128/spectrum.02715-23)
Supplement: Figure S2 — 16S beta diversity analyses. [file spectrum.02715-23-s0002.pdf]

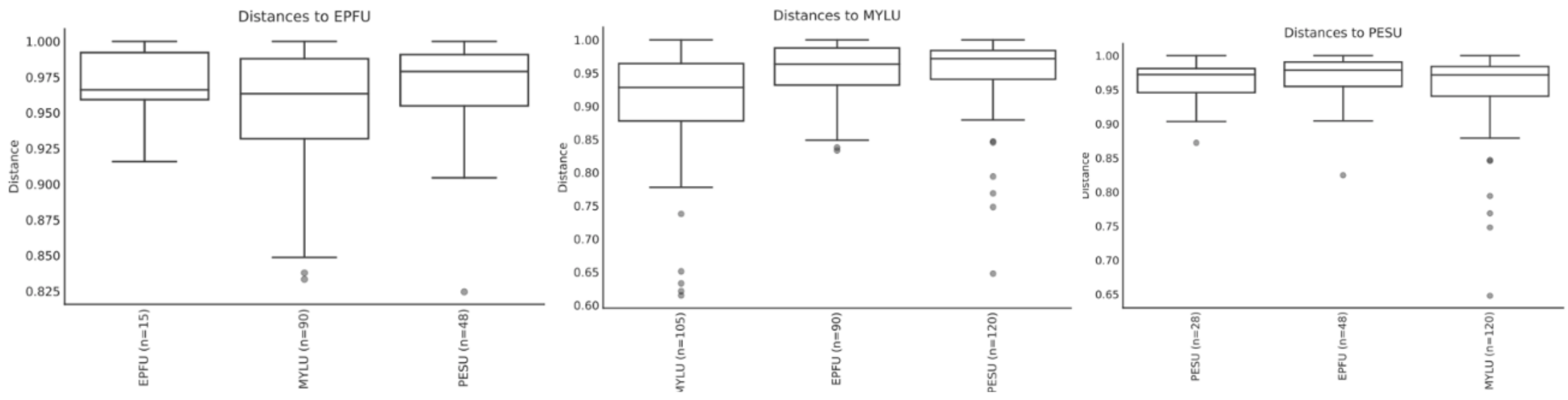

Figure S2. Bacterial beta-diversity analyses between Pd-negative *Eptesicus fuscus*, *Myotis lucifugus*, and *Perimyotis subflavus*. Jaccard distance matrices showed that when measuring bacterial species presence and absence *E. fuscus* and *P. subflavus* were the most similar in bacterial community composition ($p = 0.19$), while *P. subflavus* and *M. lucifugus* were the most dissimilar in bacterial community composition ($p = 0.02$).
