# Supplementary material for: White-nose syndrome restructures bat skin microbiomes
Source: Microbiol Spectr. 2023 Oct 27;11(6):e02715-23. doi: 10.1128/spectrum.02715-23 (PMC10714735; doi:10.1128/spectrum.02715-23)
Supplement: Figure S3 — ITS beta diversity analyses. [file spectrum.02715-23-s0003.pdf]

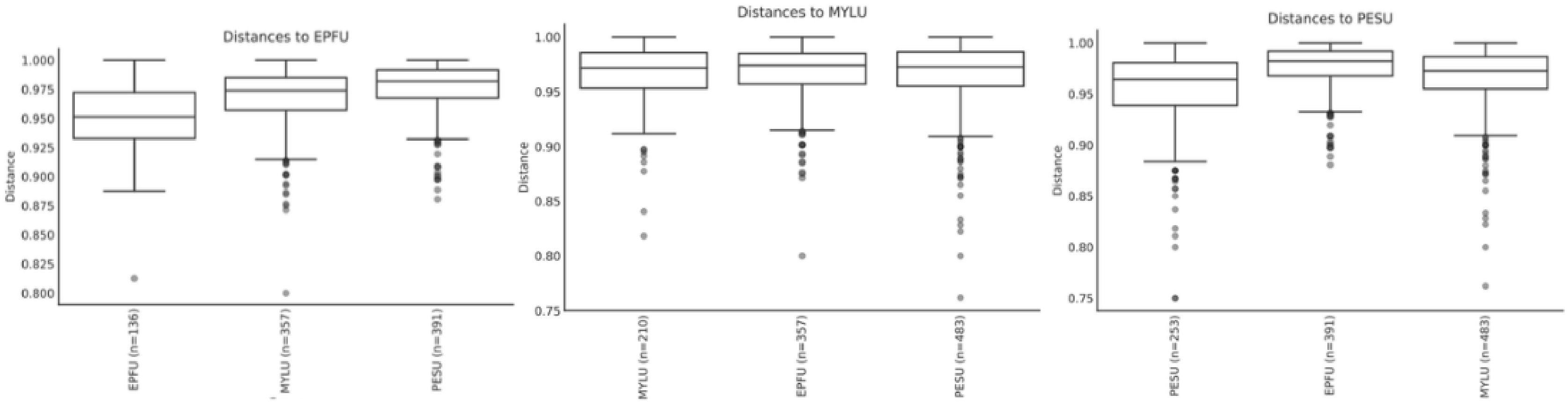

Figure S3. Fungal beta-diversity analyses between Pd-negative *Eptesicus fuscus*, *Myotis lucifugus*, and *Perimyotis subflavus*. Jaccard distance matrices for fungal community dissimilarity showed high levels of fungal community dissimilarity between all three species (*E. fuscus* and *M. lucifugus*, p = 0.001; *E. fuscus* and *P. subflavus*, p = 0.001; *M. lucifugus* and *P. subflavus*, p = 0.008).
