# Supplementary material for: White-nose syndrome restructures bat skin microbiomes
Source: Microbiol Spectr. 2023 Oct 27;11(6):e02715-23. doi: 10.1128/spectrum.02715-23 (PMC10714735; doi:10.1128/spectrum.02715-23)
Supplement: Figure S4 — 16S and ITS principal coordinate plots. [file spectrum.02715-23-s0004.pdf]

(a)

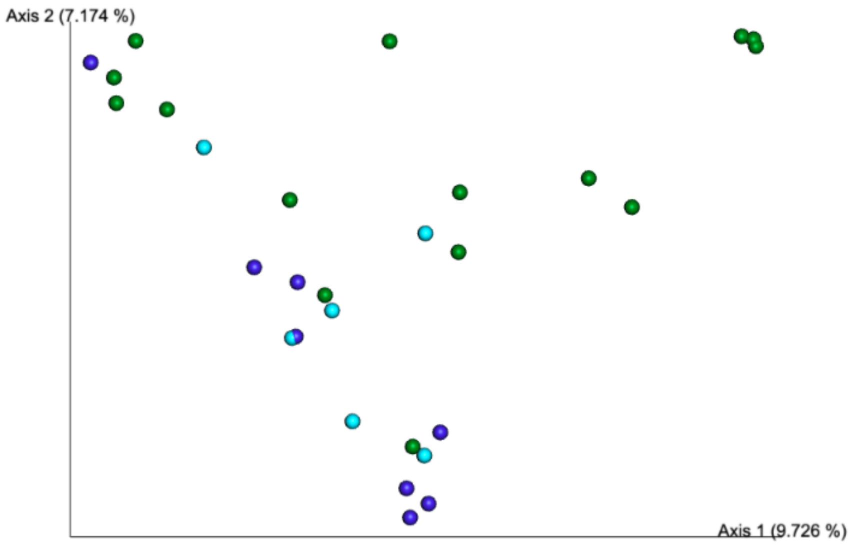

(b)

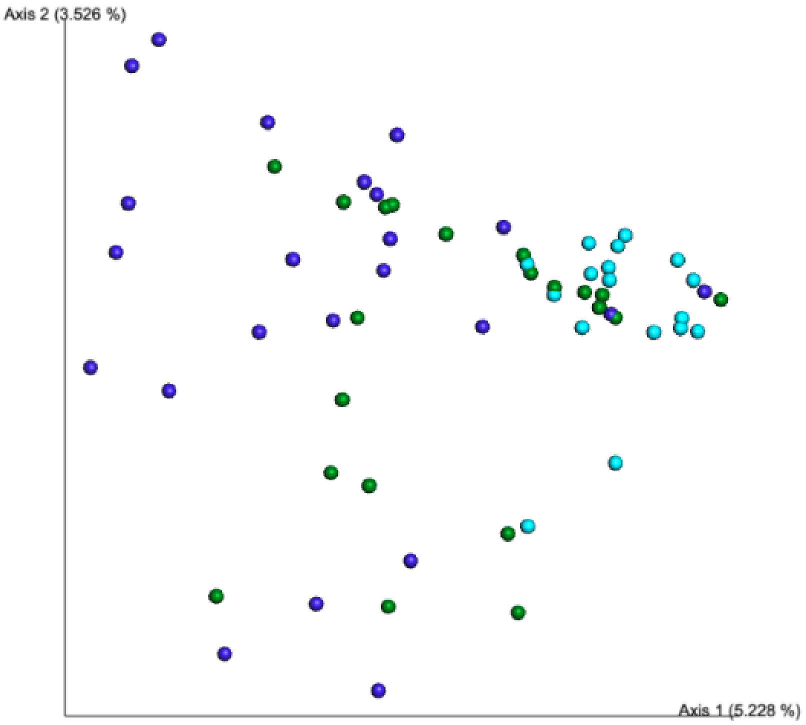

Figure S4. Bacterial (a) and fungal (b) principal coordinate plots between Pd-negative *Eptesicus fuscus* (light blue dots), *Myotis lucifugus* (green dots), and *Perimyotis subflavus* (dark blue dots). Points represent individual samples which are arranged in space based on their coordinates on the first two principal components.
