# Supplementary figures and images for: White-nose syndrome restructures bat skin microbiomes
Source: Microbiol Spectr. 2023 Oct 27;11(6):e02715-23. doi: 10.1128/spectrum.02715-23 (PMC10714735; doi:10.1128/spectrum.02715-23)

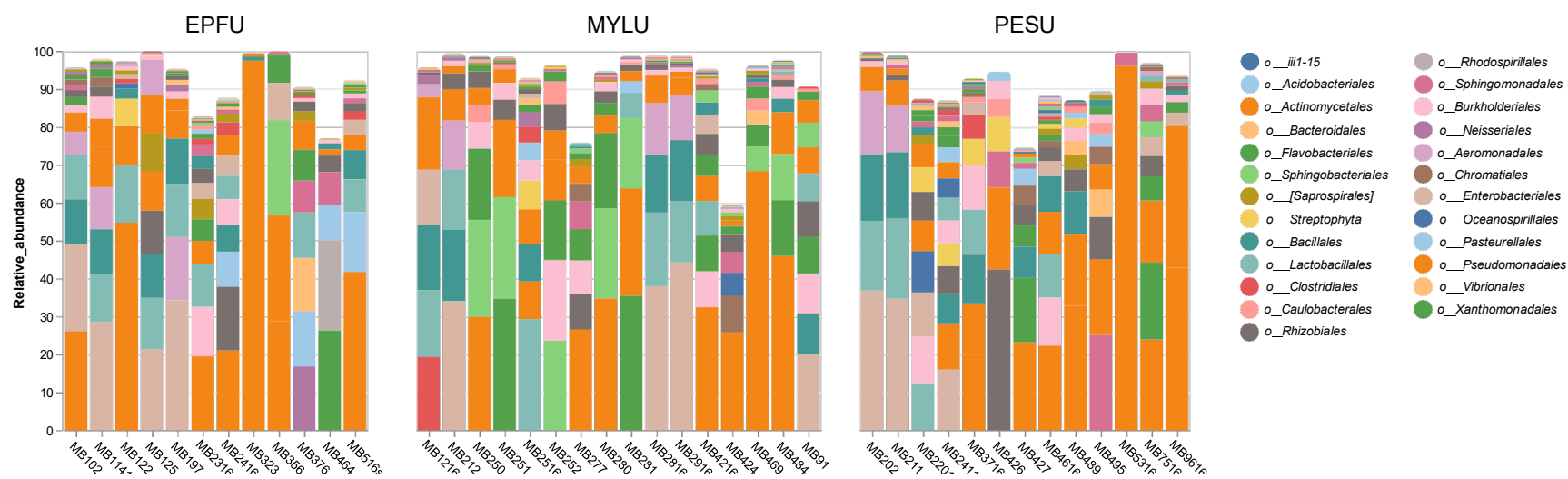

Figure S5. Bacterial taxonomic bar plot.

Supplement: Figure S5 — 16S taxonomic bar plot. [file spectrum.02715-23-s0005.pdf]
