# Supplementary material for: White-nose syndrome restructures bat skin microbiomes
Source: Microbiol Spectr. 2023 Oct 27;11(6):e02715-23. doi: 10.1128/spectrum.02715-23 (PMC10714735; doi:10.1128/spectrum.02715-23)
Supplement: Figure S7 — Shannon diversity analysis. [file spectrum.02715-23-s0007.pdf]

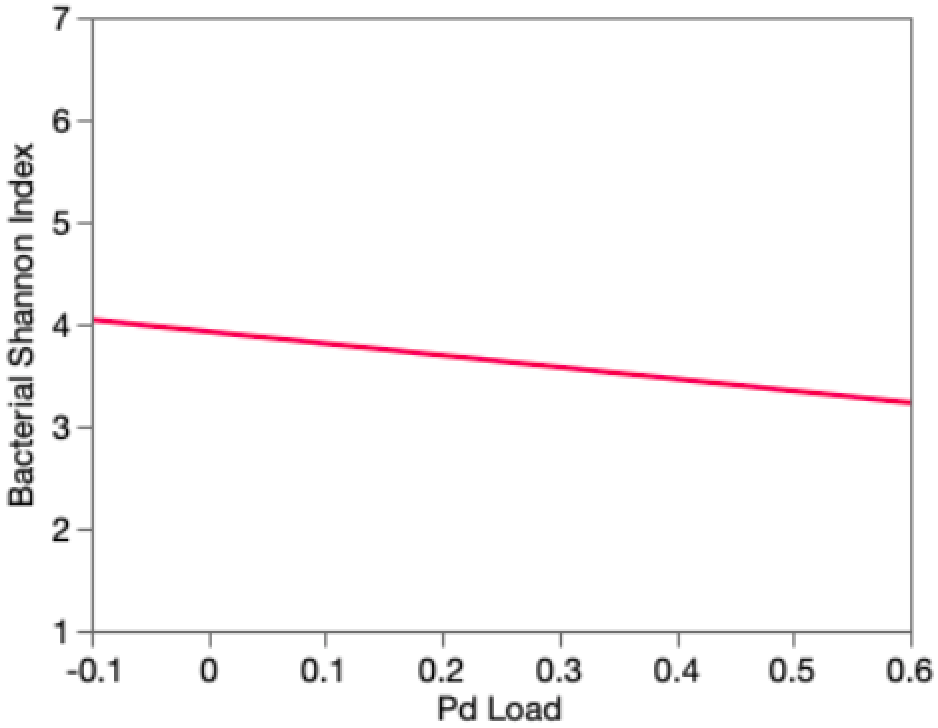

Figure S7. The effect of Pd load on Shannon diversity in *Myotis lucifugus*. Results indicate that increases in Pd load correlated with a decrease in bacterial diversity.
