# Supplementary material for: White-nose syndrome restructures bat skin microbiomes
Source: Microbiol Spectr. 2023 Oct 27;11(6):e02715-23. doi: 10.1128/spectrum.02715-23 (PMC10714735; doi:10.1128/spectrum.02715-23)
Supplement: Figure S8 — 16S differential abundance. [file spectrum.02715-23-s0008.pdf]

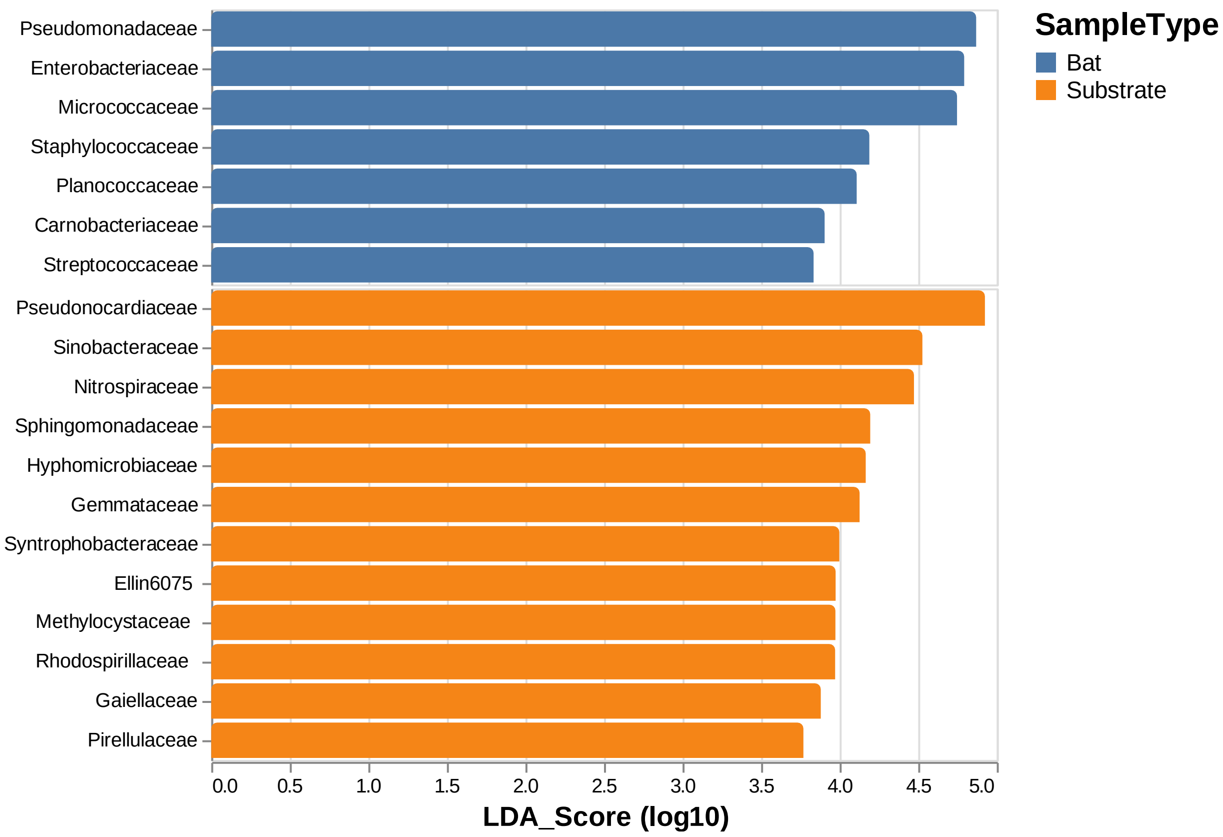

Figure S8. Bacterial differential abundance between bat and substrate samples. Bacterial diversity was significantly higher on substrates than on bats with 17 abundant bacterial families represented in the combined substrate samples and only four abundant bacterial families represented in combined bat samples.
