# Supplementary material for: White-nose syndrome restructures bat skin microbiomes
Source: Microbiol Spectr. 2023 Oct 27;11(6):e02715-23. doi: 10.1128/spectrum.02715-23 (PMC10714735; doi:10.1128/spectrum.02715-23)
Supplement: Figure S6 — ITS taxonomic bar plot. [file spectrum.02715-23-s0006.pdf]

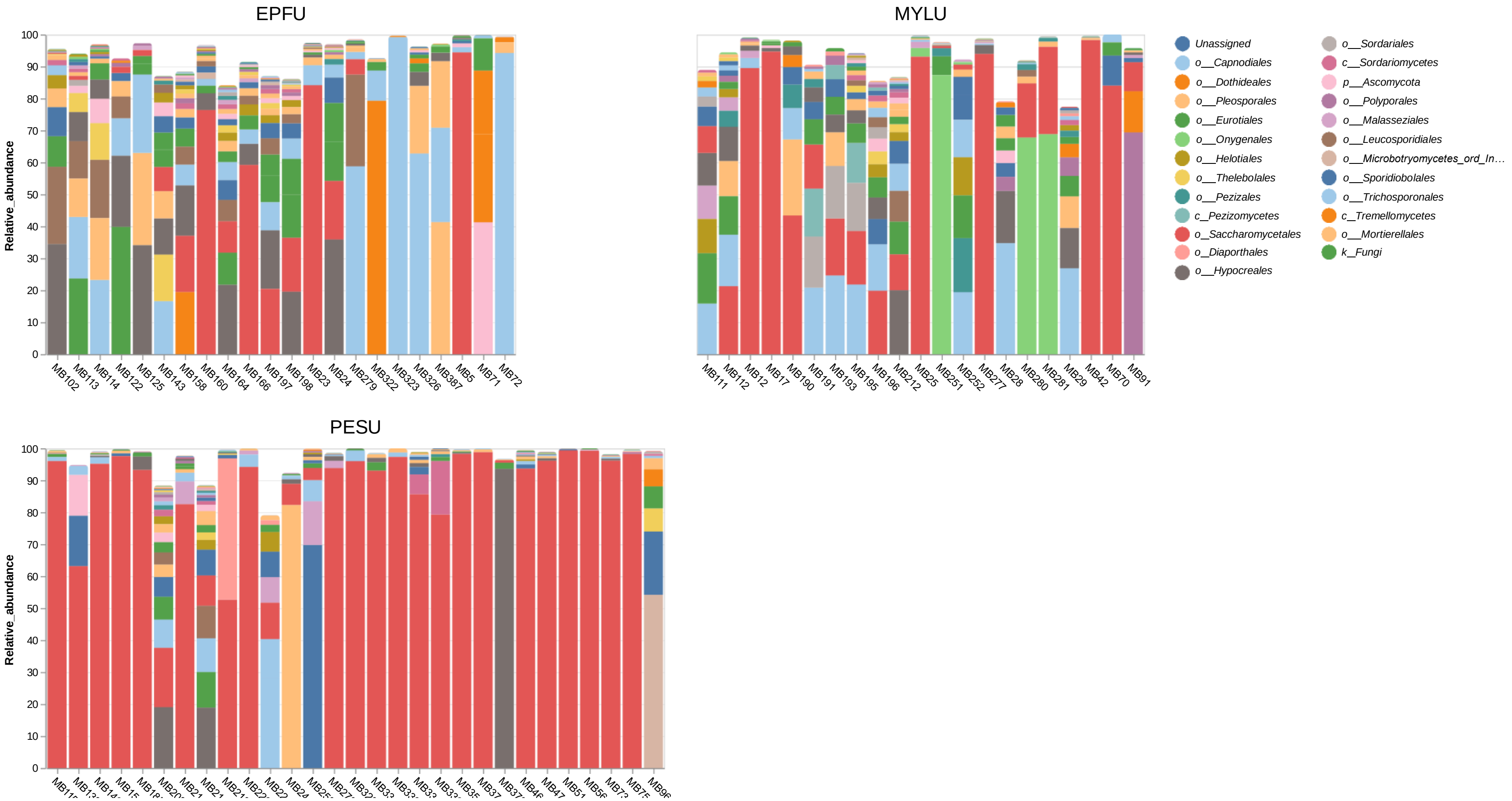

Figure S6. Fungal taxonomic bar plot.
